# Sarcopenia Is Associated With Severe Treatment Toxicity and Mortality in Older Adults With Advanced Cancer: A Secondary Analysis

**DOI:** 10.1002/jcsm.70388

**Published:** 2026-09-24

**Authors:** Lindsey J. Mattick, Mostafa Mohammed, Chin‐Shang Li, Rachael G. Tylock, Kah Poh Loh, Marie A. Flannery, Luke J. Peppone, Po‐Ju Lin, Marcus D. Goncalves, Bette J. Caan, Elizabeth M. Cespedes Feliciano, Karteek Popuri, Mirza Faisal Beg, James D. Bearden, Jeffrey Berenberg, Khalil Katato, Karen M. Mustian, Supriya G. Mohile, Richard F. Dunne

**Affiliations:** ^1^ Division of Supportive Care in Cancer, Department of Surgery, School of Medicine and Dentistry University of Rochester Medical Center Rochester New York USA; ^2^ Department of Public Health Sciences, School of Medicine and Dentistry University of Rochester Medical Center Rochester New York USA; ^3^ Division of Hematology and Oncology, Department of Medicine, School of Medicine and Dentistry University of Rochester Medical Center Rochester New York USA; ^4^ School of Nursing University of Rochester Medical Center Rochester New York USA; ^5^ Department of Medicine New York University Grossman School of Medicine New York New York USA; ^6^ Division of Research Kaiser Permanente Northern California Pleasanton California USA; ^7^ Department of Computer Science Memorial University of Newfoundland St. John's NL Canada; ^8^ School of Engineering Science Simon Fraser University Burnaby BC Canada; ^9^ Upstate Carolina Consortium Community Oncology Research Program (UPSTATE) NCORP Spartanburg South Carolina USA; ^10^ Hawaii NCORP Honolulu Hawaii USA; ^11^ Michigan Cancer Research Consortium (MCRC) NCORP Ann Arbor Michigan USA

**Keywords:** cancer, geriatric oncology, sarcopenia, treatment tolerability

## Abstract

**Background:**

Sarcopenia, a loss of muscle strength, quantity and performance that occurs primarily from ageing or secondary to disease, is hypothesized to worsen treatment outcomes in patients with cancer. Most published studies, however, focus solely on muscle quantity in patients under 70. Here, we evaluate the association between sarcopenia, defined as both a loss of strength and muscle quantity, and treatment tolerability in older adults receiving cancer treatment.

**Methods:**

This secondary analysis examines data from a randomized clinical trial evaluating a geriatric assessment intervention (NCT02054741). Older adults (≥ 70 years) with advanced cancer starting a new treatment regimen were recruited from community oncology practices across the United States. We analysed data from 161 participants in the usual care arm with baseline standard of care computed tomography (CT) scans. Sarcopenia was defined by reduced strength (chair stand > 16.7 s for five rises) and either a reduction in muscle quantity (skeletal muscle index [SMI] < 41 cm^2^/m^2^ women, < 43 cm^2^/m^2^ men with BMI < 24.9 kg/m^2^ or < 53 cm^2^/m^2^ men with BMI ≥ 25 kg/m^2^) or muscle quality. Sarcopenia was considered severe when the above criteria were met along with impaired physical performance (TUG > 13.5 s). Treatment toxicity was assessed via Common Terminology Criteria for Adverse Events (CTCAE) v4.0.

**Results:**

Among 161 participants (mean age: 76.6 ± 4.7; 61% male, 94% non‐Hispanic white), 57% (91/161) met criteria for sarcopenia, of which 59% (54/91) were considered severe. Sarcopenia was associated with deficits in multiple geriatric assessment domains (nutrition, activities of daily living, instrumental activities and cognition; *p* < 0.05). In multivariable logistic regression and Cox proportional hazards regression analyses, patients with sarcopenia were more likely to experience severe non‐hematologic toxicities (Grades 3–5; OR = 2.24, 95% confidence interval [CI]: 1.12–4.47). In participants receiving first‐line therapy, sarcopenia was associated with worse 1‐year survival (46% survival [95% CI: 35%–55%]; HR = 2.12 [95% CI: 1.14–3.93]; 49 events) compared to those without sarcopenia (60% survival [95% CI: 48%–70%]; 28 events). SMI alone was not significantly associated with severe nonhematologic toxicity or survival.

**Conclusion:**

Sarcopenia is highly prevalent in older patients with cancer and is associated with severe toxicity and mortality. Our findings highlight the importance of assessing muscle strength, quantity, quality and performance when evaluating older adults with cancer.

****Trial Registration:** ClinicalTrials.gov NCT02054741:**

## 1 Introduction

More than half of all patients with cancer are over the age of 65 [1], yet older adults, especially those over 75, remain underrepresented in treatment trials [2]. Perceived poor performance status by clinicians and the presence of limiting comorbid conditions have been shown to contribute to low participation in this population [3]. Regardless, this lack of representation hinders our understanding of treatment tolerability and the risks associated with serious toxicities in this vulnerable and growing demographic. Severe and persistent cancer treatment toxicities can lead to hospitalization or the need for additional interventions to manage side effects, ultimately increasing the healthcare burden and diminishing quality of life (QOL) [4, 5]. These toxicities can also be dose‐limiting, compromising survival outcomes.

Evidence suggests that cancer treatment toxicities may be worsened by sarcopenia, an ageing‐related condition marked by the loss of muscle quantity and function [6, 7]. Muscle strength, quantity and quality decline with advanced age as ageing results in atrophy of muscle fibres (mainly type II fibres) [8], a reduction in motor units and impairment of the neuromuscular junction [9] and a decrease in protein synthesis, which ultimately inhibits the ability to build or maintain mass [10]. While there is evidence that lifestyle intervention (i.e., improved nutrition and exercise) may attenuate a loss of muscle quantity in older adults with sarcopenia [11, 12], there is little randomized evidence that these benefits can be translated to older patients with cancer receiving cytotoxic chemotherapy [13, 14, 15].

Sarcopenia affects up to 22% of older adults worldwide and contributes to unhealthy ageing by impairing an individual's ability to maintain functional independence and QOL [16, 17]. The condition increases the risk for fractures, functional decline, hospitalizations and mortality [18].

Sarcopenia can occur physiologically as a normal part of ageing or inactivity [19], but can also be pathological, arising from conditions like chronic obstructive pulmonary disease (COPD) or cancer [20, 21]. Secondary sarcopenia that occurs in the setting of cancer is also commonly referred to as cancer cachexia. Significant overlap exists between these two wasting conditions. Cachexia, like sarcopenia, is also defined by a loss of muscle quantity and physical function but can also involve loss of fat mass and is highlighted by a pro‐inflammatory, catabolic state; both are associated with poor cancer and cancer treatment outcomes. Older adults with advanced cancer can be experiencing both sarcopenia and cachexia, which both may be compounded by direct and indirect effects of cancer treatment [22, 23, 24]. As such, it may be difficult to discern which component, and to what degree each wasting condition contributes to changes in body composition, function and negative outcomes. As the scope of this research involves only the geriatric oncology population, we utilize the term sarcopenia as a broader muscle condition among older patients with cancer.

The term sarcopenia has commonly been used by cancer researchers to refer solely to the loss of muscle quantity without considering deficits in strength or performance. However, in older adults, the literature suggests that examining muscle quantity alone in older patients with cancer is insufficient, as outcomes in this population may be more closely aligned with muscle strength and performance [25]. Moreover, muscle quantity is not a reliable proxy for muscle function (strength and performance), as the relationship between quantity and function is not linear [26]. Recognizing this limitation, all major clinical definitions of sarcopenia now incorporate measures of low muscle quantity (assessed by both muscle quantity and quality), as well as impaired strength and decreased performance [27, 28, 29]. Combining these measures identifies a population of adults with functional limitations who are likely to benefit from interventions focused on the muscle. Though a systematic review found that pretreatment sarcopenia, mostly defined by the skeletal muscle index (SMI) alone, may be associated with poor cancer outcomes, including chemotherapy toxicity, almost all studies examined were retrospective, lacked information on muscle strength and performance or were limited to small cohorts of patients under the age of 70 [7, 30].

The impact of clinically defined sarcopenia on older adults across cancer types, particularly within frail populations, remains understudied. These data were then paired with detailed documentation of clinician‐reported adverse events using the Common Terminology Criteria for Adverse Events (CTCAE) v.4 in patients receiving systemic therapy for advanced cancer [31]. In a large US nationwide cohort of older adults with advanced cancer, we aimed to evaluate whether clinically defined sarcopenia and measures of muscle quantity, strength and performance at baseline were associated with treatment toxicity and 1‐year mortality.

## 2 Methods

### 2.1 Study Design

The Geriatric Assessment for Patients (GAP70+) study (ClinicalTrials.gov identifier: NCT02054741) is a multicentre, nationwide, cluster‐randomized clinical trial (RCT) conducted across 39 community oncology clinics affiliated with the University of Rochester Cancer Center (URCC) NCI Community Oncology Research Program (NCORP) Research Base [32]. This study was approved by the Institutional Review Boards (IRBs) at all participating sites. All participants provided written informed consent at enrollment for all study activities and review of clinical, pathological laboratory and radiographic data. We collected abdominal and pelvic computed tomography (CT) or positron emission tomography‐computed tomography (PET/CT) scans from enrolled participants, obtained as part of standard care; baseline scans were to be within 8 weeks prior to or 4 weeks after study enrollment to be considered eligible for analysis.

### 2.2 Study Participants

**FIGURE 1:**
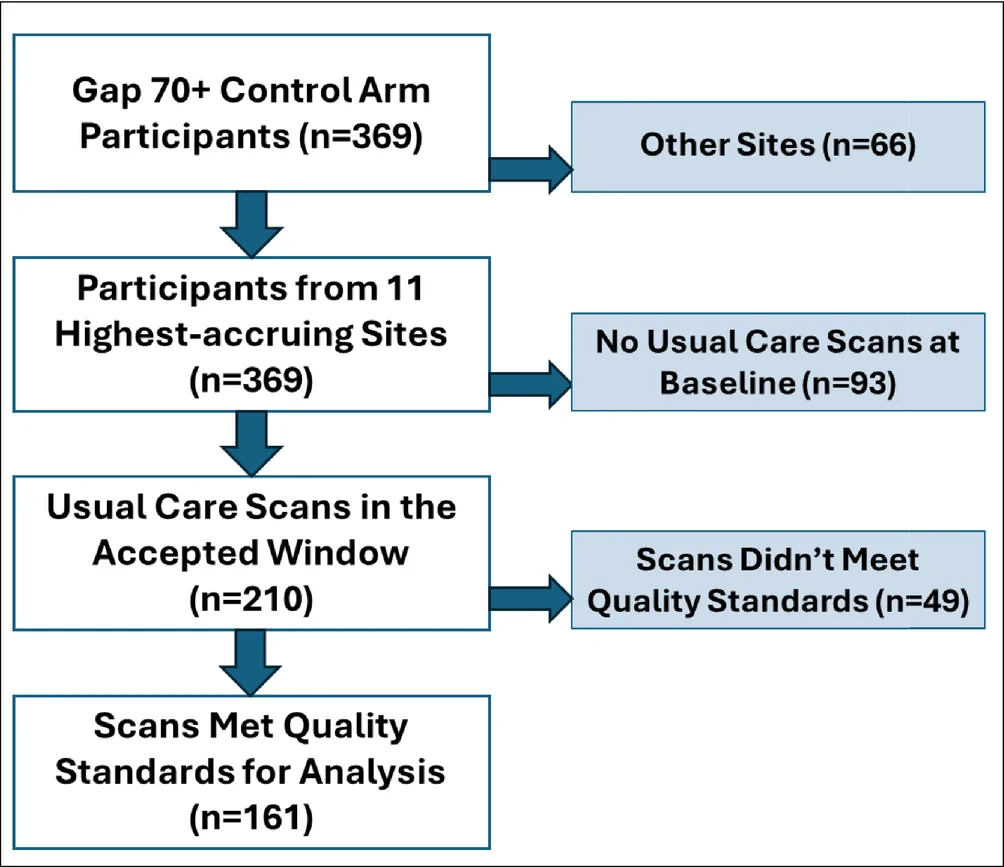
CONSORT Diagram.

Eligible participants for the GAP70 + trial were aged 70 years or older, diagnosed with an incurable stage III/IV solid tumour or lymphoma, impaired in at least one GA domain (e.g., cognition, social support, psychological status) and scheduled to initiate a new systemic cancer treatment regimen with a high risk of toxicity (defined as cytotoxic chemotherapy or nine other agents with a severe toxicity prevalence of > 50%) within 4 weeks of baseline registration. In total, 718 participants were recruited for the GAP70 + trial. This sarcopenia analysis included usual care participants only. As physicians of intervention arm participants were provided with a GA summary that may have influenced chemotherapy treatment decisions, intervention arm participants were excluded. Of the 363 patients in the usual care arm, scans were collected from participants at 11 of the highest enrolling sites (*n* = 303). Among them, 210 participants had imaging scans within the required time window, and 161 had scans that met quality standards for analysis (Figure 1).

### 2.3 Measures

Socio‐demographic variables, including age, sex (male, female), race (white, black and others), education (less than high school, high school graduate and some college or more) and income (≤ $50 000 and > $50 000), were collected from patient‐reported questionnaires at baseline. Clinical information, including cancer type (gastrointestinal, lung, breast and others), cancer stage (stages III and IV), line of palliative treatment (first line and second line or more) and physician‐reported Karnofsky Performance Score (40–60, 70–80 and 90–100), was abstracted from the clinical record.

A GA was administered to all participants as part of the GAP70 + study procedures. The GA includes eight different domains to assess comorbidity, physical performance (including a five‐time chair‐stand test and a timed up and go [TUG] test), cognition, instrumental social support, polypharmacy, psychological health and nutritional status.

We performed manual anatomical landmarking to identify the mid‐L3 lumbar vertebrae image slice for each individual CT scan. The validated automatic segmentation software Data Analysis Facilitation Suite (DAFS) V3 by Voronoi Health Analytics Inc. (https://www.voronoihealthanalytics.com/) was used to perform automatic segmentation of Mid‐L3 CT slice images into skeletal muscle and adipose tissue (subcutaneous and visceral) compartments, generating quantitative image‐derived body composition metrics [33, 34]. Each CT scan underwent rigorous quality control at three different points of data processing: at initial receipt of raw CT images, after CT scans underwent manual anatomical landmarking and at the completion of DAFS auto‐segmentation prior to data output.

Sarcopenia definitions were established by the European Working Group on Sarcopenia in Older People (EWGSOP2) using three criteria: (1) reduced muscle strength, (2) reduced quantity and quality and (3) reduced physical performance [6]. Reduced muscle strength was defined as a five‐time chair‐stand time of > 16.7 s for five rises [35]. Reduced muscle quantity was defined as a SMI < 41 cm^2^/m^2^ for women and < 43 cm^2^/m^2^ for men with a BMI < 24.9 kg/m^2^ or < 53 cm^2^/m^2^ for men with a BMI ≥ 25 kg/m^2^ [36]. Reduced muscle quality was defined as a Hounsfield Unit (HU) measure of < 41 HU for those with a BMI < 24.9 kg/m^2^ or < 33 HU for those with a BMI ≥ 25 kg/m^2^ [36]. Reduced physical performance was indicated by a TUG test time of > 13.5 s [37]. Sarcopenia was defined as meeting both criterion one (reduced muscle strength) and criterion two (for either reduced muscle **quantity** or quality). Severe sarcopenia was classified as meeting all three criteria.

Severe toxicity was defined as Grades 3–5 adverse events obtained via the physician‐reported Common Terminology for Adverse Events (CTCAE) v4.0. A Research Base team, blinded to study arm, led by an oncologist (MM), reviewed toxicity grading by comparing data forms with the medical record. If discrepancies were identified, practice staff reviewed and resolved them. Mortality was assessed at 1 year following treatment initiation.

### 2.4 Statistical Analysis

Descriptive statistics were used to summarize baseline characteristics. Continuous or numerical variables were reported as means and standard deviations, as well as medians, minima and maxima. Categorical variables were presented as frequencies and percentages. Differences between those with sarcopenia or severe sarcopenia compared to those with no sarcopenia were assessed using the Chi‐squared test or Fisher's exact test, as appropriate for nominal categorical variables, and the Wilcoxon rank‐sum test for continuous or numerical variables.

Logistic regression was performed to assess the relationship of Grades 3–5 toxicities with sarcopenia, severe sarcopenia and continuous measures of muscle quantity, strength and performance. Models adjusted for age (continuous), tumour site (categorical variable; GI, lung, and other), sex (male/female), impaired comorbidities (more than three comorbidities) and treatment type (categorical variable; single‐agent chemotherapy alone, multiple‐agent chemotherapy alone, other nonchemotherapy agents alone and chemotherapy with other agents).

Participants were followed from study enrollment to death, disenrollment or end of follow‐up at 1 year, whichever came first. For univariable survival analysis, the Kaplan–Meier method was used to estimate the survival curve for each group (low vs. preserved SMI, sarcopenia vs. no sarcopenia, severe sarcopenia vs. no sarcopenia). The log‐rank test was employed to compare survival curves between groups [38]. Cox proportional hazards regression was performed to estimate hazard ratios (HRs) and 95% confidence intervals (CIs) for 1‐year mortality, adjusting for age (continuous), tumour site (categorical variable; GI, lung and other), sex (male/female), impaired comorbidities (more than three comorbidities), treatment type (categorical variable: chemotherapy + other agents, chemotherapy only and other‐agents‐only) and prior chemotherapy (yes/no) [39]. A *p*‐value of < 0.05 was considered statistically significant. All analyses were performed using SAS version 9.4 (SAS Institute Inc., Cary, NC).

## 3 Results

Among the 161 participants included in this study (Figure 1), the mean age was 76.6 years, 95.7% were non‐Hispanic white, 60.9% were male and over half had gastrointestinal (34.8%) or lung cancer (23.6%). Overall, 68.3% (110/161) met the definition of low muscle quantity; 56.5% (91/161) met the clinical definition of sarcopenia, the majority of whom (59%; 54/91) were categorized as having severe sarcopenia. Participants with sarcopenia were significantly more likely to have diabetes (32% vs. 17%) and were, on average, 2 years older (77.5 vs. 75.5 years) than those without sarcopenia (Table 1). Similarly, those with the most severe form of sarcopenia were significantly more likely to be over the age of 80, compared to those without sarcopenia.

**TABLE 1:** Selected demographic and clinical characteristics of the study sample according to sarcopenia (*N* = 161).

Mean ± SD (median, min, max)	No sarcopenia (*n* = 70)	Sarcopenia (*n* = 91)	Sarcopenia vs. no sarcopenia *p*‐value	Severe sarcopenia (*n* = 54)	Severe sarcopenia vs. no sarcopenia *p*‐value
Age (years)	75.5 ± 4.3 (75, 70, 88)	77.5 ± 4.8 (77, 70, 92)	0.008^a^	77.2 ± 5.0 (77, 70, 92)	0.059
*N* (%)					
Categorized age			0.011^a^		0.037
70–79	60 (86%)	62 (68%)		38 (70%)	
80–89	10 (14%)	28 (31%)		15 (28%)	
≥ 90	0	1 (1%)		1 (2%)	
Education			0.714^a^		0.920
< High school	9 (13%)	16 (18%)		7 (13%)	
High school graduate	25 (36%)	29 (32%)		20 (37%)	
≥ Some college	36 (51%)	46 (50%)		27 (50%)	
Sex			0.436^b^		0.567
Male	45 (64%)	53 (58%)		32 (59%)	
Female	25 (36%)	38 (42%)		22 (41%)	
Race			0.480^c^		0.326^c^
Non‐Hispanic White	67 (96%)	87 (96%)		50 (92%)	
African American	3 (4%)	2 (2%)		2 (4%)	
Others	0	2 (2%)		2 (4%)	
Tumour site			0.480^b^		0.418^b^
GI	21 (30%)	35 (38%)		23 (43%)	
Lung	17 (24%)	21 (23%)		12 (22%)	
Breast	10 (14%)	7 (8%)		4 (7%)	
Other	22 (32%)	28 (31%)		15 (28%)	
Diabetes mellitus			0.034^b^		0.155^b^
No	58 (83%)	62 (68%)		39 (72%)	
Yes	12 (17%)	29 (32%)		15 (28%)	
Reduced RDI at Cycle 1			0.082^b^		0.252^b^
No	54 (77%)	58 (64%)		36 (67%)	
Yes	16 (23%)	32 (35%)		17 (32%)	
Missing	0	1		1	
Cancer treatment type			0.192^b^		0.114^b^
Chemotherapy alone: Single	17 (24%)	13 (14%)		6 (11%)	
Chemotherapy alone: Multiple	32 (46%)	45 (50%)		29 (54%)	
Chemotherapy and nonchemotherapy agents	15 (21%)	17 (19%)		9 (17%)	
Nonchemotherapy alone (e.g., targeted agents)	6 (9%)	16 (17%)		10 (18%)	

Abbreviations: GI, gastrointestinal; RDI, relative dose intensity.

^a^ Wilcoxon rank‐sum test.

^b^ Chi‐squared test.

^c^ Fisher’s exact test.

Among participants with sarcopenia, we report a higher prevalence of baseline functional impairment, including dependence in activities of daily living (ADLs), and instrumental ADLs (IADLs), more recent falls and poorer cognition compared to those without sarcopenia (all *p* < 0.05), as shown in Table 2. Participants with severe sarcopenia were significantly more likely to report impairments in all five key geriatric assessment (GA) tests compared to those without sarcopenia including nutrition (76% vs. 53%; *p* = 0.008), ADL dependence (43% vs. 14%; *p* < 0.001), IADL dependence (74% vs. 40%; *p* < 0.001), recent falls (26% vs. 11%; *p* = 0.036) and impaired cognition (50% vs. 29%; *p* = 0.015).

**TABLE 2:** Associations of low SMI, sarcopenia and severe sarcopenia with key geriatric assessment domains/tests (*N* = 161).

	Low SMI *n* = 110 *N* (%)	Preserved SMI *n* = 51 *N* (%)	Low vs. preserved *p*	Sarcopenia *n* = 91 *N* (%)	No sarcopenia *n* = 70 *N* (%)	Sarcopenia vs. no sarcopenia *p*‐value	Severe sarcopenia *n* = 54 *N* (%)	Severe sarcopenia vs. no sarcopenia *p*‐value
Nutrition (MNA)								0.008
Impaired	74 (67%)	21 (44%)	0.002	58 (64%)	37 (53%)	0.164	41 (76%)	
Not impaired	36 (33%)	30 (56%)		33 (36%)	33 (47%)		13 (24%)	
ADL								< 0.001
Dependent	29 (26%)	10 (20%)	0.073	26 (29%)	10 (14%)	0.031	23 (43%)	
Independent	81 (74%)	41 (80%)		65 (71%)	60 (86%)		31 (57%)	
IADL								< 0.001
Dependent	58 (53%)	23 (45%)	0.368	53 (58%)	28 (40%)	0.022	40 (74%)	
Independent	52 (47%)	28 (55%)		38 (42%)	42 (60%)		14 (26%)	
Falls								0.036
Recent fall	22 (20%)	12 (24%)	0.954	24 (26%)	8 (11%)	0.019	14 (26%)	
No falls	88 (80%)	39 (76%)		67 (74%)	62 (89%)		40 (74%)	
Cognition								0.015
Impaired	35 (32%)	21 (41%)	0.246	35 (38%)	20 (29%)	0.190	27 (50%)	
Not impaired	75 (68%)	30 (59%)		56 (62%)	50 (71%)		27 (50%)	

*Note:* All *p*‐values obtained using chi‐squared test.

Abbreviations: ADL, activities of daily living; IADL, instrumental activities of daily living; MNA, Mini Nutrition Assessment.

The prevalence of experiencing severe (Grades 3–5) toxicity during the first 3 months of therapy, as measured by CTCAE, is shown in Table 3. Severe overall toxicity prevalence did not significantly differ according to whether participants met the definition of sarcopenia (79% vs. 71%; *p* = 0.272). However, severe nonhematologic toxicities specifically were significantly more prevalent among participants with sarcopenia compared to those without sarcopenia (64% vs. 44%; *p* = 0.022). In multivariable logistic regression analyses, participants with sarcopenia were more than twice as likely to experience severe nonhematologic toxicities (odds ratio [OR] = 2.24; 95% CI: 1.12–4.46). A detailed breakdown of which nonhematologic toxicities participants with sarcopenia experienced is available in Table S1.

**TABLE 3:** Odds of Grades 3–5 treatment‐related toxicities according to sarcopenia status and measures of muscle mass, strength and physical function (*N* = 159).

	Any Grades 3–5 toxicity				Any Grades 3–5 nonhematologic toxicity			
	Yes (%)	No (%)	*p*	Odds ratio (95% CI)^a^	Yes %	No %	*p*	Odds ratio (95% CI)^a^
Sarcopenia	79%	21%	0.272	1.57	64%	36%	0.022	2.24
No sarcopenia	71%	29%		(0.70–3.49)	44%	56%		(1.12–4.47)
Severe sarcopenia	74%	26%	0.690	1.20	57%	43%	0.130	1.84
No sarcopenia	71%	29%		(0.49–2.91)	44%	56%		(0.84–4.03)
	Yes Mean (SD)	No Mean (SD)		Odds ratio (95% CI)*	Yes Mean (SD)	No Mean (SD)		Odds ratio (95% CI)^a^
SMI (cm^**2**^/m^**2**^)	43.1 ± 8.9	41.9 ± 7.7	0.159	1.04 (0.99, 1.09)	42.7 ± 9.2	42.9 ± 7.9	0.929	1.00 (0.96, 1.05)
TUG (seconds)	12.9 ± 4.5	12.2 ± 3.7	0.276	1.07 (0.95, 1.20)	12.4 ± 3.8	13.1 ± 4.9	0.359	0.96 (0.89, 1.05)
5× chair stand (seconds)	17.7 ± 7.1	15.5 ± 4.3	0.128	1.09 (0.98, 1.21)	18.7 ± 7.9	15.4 ± 3.8	0.007	1.15 (1.04, 1.27)

^a^ Adjusted for age, tumour site, gender, impaired comorbidities, treatment type, prior chemo and standard of care.

No significant differences in the prevalence of severe overall toxicities or nonhematologic toxicities were observed when comparing participants with severe sarcopenia to those with no sarcopenia (74% vs. 71%, *p* = 0.690; 57% vs. 44%, *p* = 0.130, respectively).

Mean measures of muscle quantity (SMI; cm^2^/m^2^), muscle strength (5× chair stand; seconds) and muscle performance (TUG; seconds) among those with and without any Grades 3–5 toxicity or any Grades 3–5 nonhematologic toxicity are presented in Table 3. There were no significant differences in these measures between participants with and without any Grades 3–5 toxicity.

However, participants who experienced Grades 3–5 nonhematologic toxicity had significantly worse muscle strength, as demonstrated by longer 5 × chair stand times (18.7 ± 7.9 vs. 15.4 ± 3.8 s; *p* = 0.007). This association remained significant after adjustment for covariates; the odds of Grades 3–5 nonhematologic toxicity increased with better muscle strength measures (OR = 1.15, 95% CI: 1.04–1.27).

**FIGURE 2:**
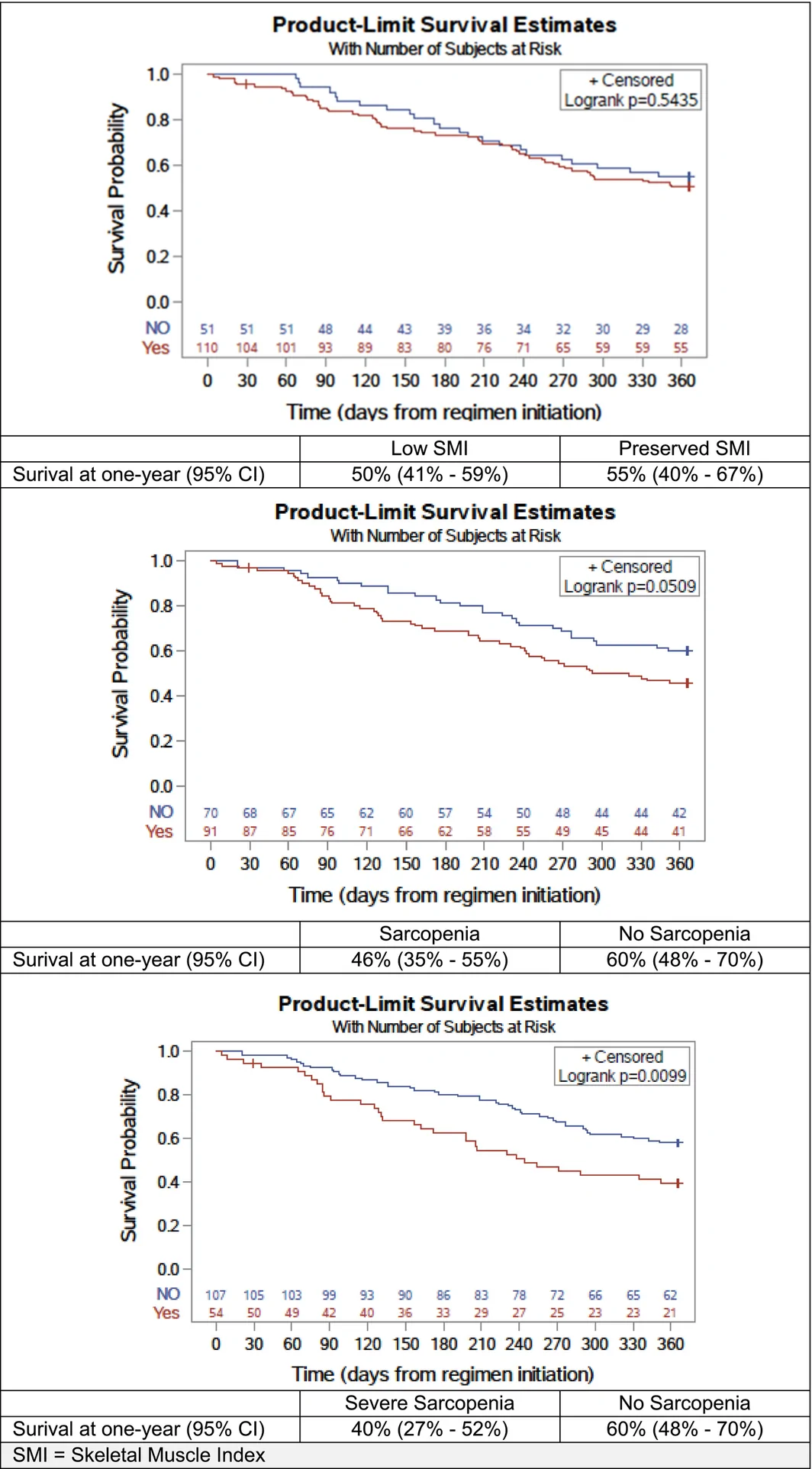
Kaplan–Meier curves. One‐year survival for low SMI, sarcopenia, and severe sarcopenia (*n* = 161).

**FIGURE 3:**
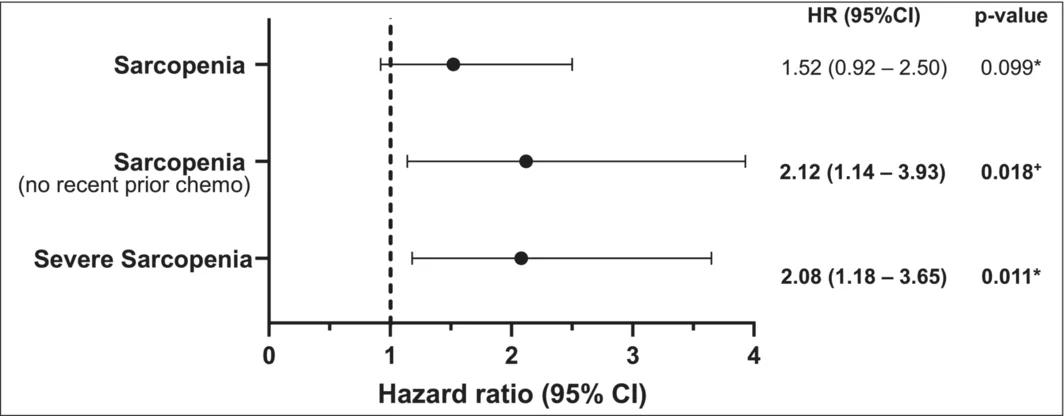
Cox proportional hazards model. One‐year survival for sarcopenia and severe sarcopenia compared to no sarcopenia (*n* = 159). All analyses compare No Sarcopenia as the referent group. * Adjusted for age, tumour site, gender, impaired comorbidities, treatment type, and prior chemo. ^+^ Adjusted for age, tumour site, gender, impaired comorbidities, and treatment type.

At 1 year follow‐up, 46% of participants with sarcopenia were alive (49 events), compared to 60% of participants without sarcopenia (28 events), indicating a trend for worse survival associated with sarcopenia (*p* = 0.051; Kaplan–Meier curves shown in Figure 2). After adjustment for age, cancer type, sex, comorbidities, treatment type and recent chemotherapy exposure, this association with survival was not statistically significant (Figure 3; HR = 1.52; 95% CI: 0.92–2.53; *p* = 0.099). However, when examining participants enrolled at the start of first‐line chemotherapy (*n* = 121), the multivariable Cox proportional hazards regression analysis indicates that sarcopenia was significantly associated with poorer 1‐year survival (HR [95% CI] = 2.12 [1.14–3.93]; *p* = 0.018). Mortality risk was even more pronounced when comparing those participants with severe sarcopenia to those without sarcopenia (1‐year survival of 40% vs. 60%; *p* = 0.010; 32 vs. 28 events) and remained statistically significant after adjusting for the covariates described above (HR [95% CI] = 2.08 [1.18–3.65]; *p* = 0.011). This mortality gap emerged early; at 3 months, nearly 21% of individuals with severe sarcopenia had died, versus 7% of their counterparts without sarcopenia (*p* = 0.086). In contrast, when evaluating SMI alone, without the functional components described in the criteria for sarcopenia, no significant association with 1‐year survival was observed (*p* = 0.544; Kaplan–Meier curves shown in Figure 2).

## 4 Discussion

This study is among the first to evaluate the risk of treatment toxicity and mortality in older patients with advanced cancer and sarcopenia using clinically accepted definitions of sarcopenia and clinical‐trial annotated toxicity outcomes. Our findings demonstrate that sarcopenia is not only highly prevalent in geriatric oncology populations but may also place older patients at higher risk for severe cancer treatment‐related toxicities and early mortality when receiving first‐line therapy.

Furthermore, our results highlight the limitations of relying solely on muscle quantity for evaluating outcomes in this population. Although many oncology studies of sarcopenia define this condition merely by muscle quantity, we found that diminished muscle quantity alone was not associated with toxicity, mortality, or impairment in most ageing‐related domains. Most notably, we find that it is muscle strength and not quantity that differentiates older patients with cancer at higher risk for poor outcomes, in contrast to what is seen in younger patients [7, 30, 40, 41]. This may be due to the fact that reduced muscle quantity is a physiologic consequence of ageing. In our analysis, classifying participants according to a consensus definition of sarcopenia which incorporated measures of muscle strength, quantity and performance was significantly associated with five key ageing‐related tests [6]. Sarcopenia was linked to a significantly increased risk of severe non‐hematologic toxicity and double the risk of mortality compared to participants without sarcopenia. The mortality risk also more than doubled in individuals with the most severe form of sarcopenia. Clinically, the impact was striking, with only 40% of participants with severe sarcopenia alive at 1 year compared to 60% without sarcopenia.

Our sarcopenia dataset is unique in that we have detailed data on the types of toxicities patients with sarcopenia experience while on chemotherapy. Our multivariable logistic regression and Cox proportional hazards regression analyses indicate that sarcopenia is associated with an increased risk of severe non‐hematologic treatment toxicity and mortality in geriatric oncology populations. This result is clinically meaningful as certain Grade 3 hematologic toxicities like anaemia, neutropenia and lymphopenia may not result in hospitalizations or distress for patients with cancer, whereas Grade 3 nonhematologic toxicities like electrolyte disturbance, fatigue, dehydration, infection and pain often do. These findings underscore important considerations of the inadequacy of considering muscle quantity alone in clinical management, particularly regarding the identification of patients most at risk for treatment‐related toxicity and mortality. While mortality is a critical concern, patients in geriatric oncology populations increasingly prioritize QOL and the avoidance of treatment‐related burdens, such as excessive toxicity [42].

To our knowledge, only one prior study in prostate cancer has evaluated treatment‐related toxicity in older adults with advanced cancer using a definition of sarcopenia that encompassed muscle strength, quantity and performance [30]. Mechanisms of sarcopenia may be different in this population given their exposure to treatments targeting androgens. Papadopoulos et al. analysed data from 110 male patients aged 65 and older with metastatic castrate‐resistant prostate cancer and reported a sarcopenia prevalence of 27%, notably lower than the 57% observed in our cohort. This discrepancy likely stems from differing diagnostic criteria: Their definition of sarcopenia required low muscle strength, reduced muscle quantity or quality and impaired physical performance, more closely aligning with the EWGSOP2 classification of severe sarcopenia used in our analysis [27]. Despite methodological differences, their findings are broadly consistent with ours: Sarcopenia was associated with poorer overall survival across the study population, with a higher risk of severe toxicity in patients receiving androgen receptor axis‐targeted therapy (ARAT). Our findings may be applied more broadly, given the range of tumour types evaluated in our study. Two recent studies among older patients (both mean age = 69 years) with GI cancer, neither of which included objective measures of strength and function, found that lower SMI was associated with increased treatment toxicity, while the association between SMI and survival was inconsistent. However, the inclusion of more heterogenous populations of younger patients (age range, 42–90 years), with early stage disease, along with the absence of standardized adverse event reporting limits the ability to compare these findings to our own [43, 44].

Our findings from a multicentre sample of older adults with advanced cancer treated in community oncology clinics nationwide provide a real‐world representation of the geriatric oncology population. It employs gold‐standard measures of muscle quantity and quality, along with rigorously collected data on muscle strength and function as part of a clinical trial, enabling classification based on an accepted consensus definition of sarcopenia. Additionally, the study incorporates detailed documentation of clinician‐reported adverse events from the CTCAE, meeting the quality standards required for a RCT.

This study has several limitations. First, the study sample consisted predominantly of non‐Hispanic white participants, which may limit the generalizability of the results to other race/ethnic groups. Second, scans and timing of scans were not mandated by the study. Strict time windows were employed for inclusion in the analysis to help mitigate this but by doing so limited the sample size. As this is a secondary, exploratory analysis evaluating multiple clinical outcomes, we did not adjust *p*‐values for multiple testing to avoid inflating Type II errors. Therefore, these findings should be interpreted as hypothesis‐generating and require confirmation in future prospective cohorts. Lastly, the treatment regimens were dictated by the participants' primary oncologists; standard dosing was not mandated in the trial.

Given the association between sarcopenia and poorer outcomes, our findings suggest clinicians should screen for sarcopenia in this population. Interestingly, those with severe sarcopenia received some of the most intensive treatments in the cohort we evaluated yet had the worst survival, raising the question of whether these individuals were overtreated and would have benefitted from a more comprehensive evaluation. For some older adults with severe sarcopenia, best supportive care may be more appropriate than chemotherapy given we found > 20% of patients in this subset died within 3 months of enrollment.

Understanding sarcopenia status in real‐time could positively influence cancer treatment decision‐making. Although CT scans are readily available in oncology clinics worldwide and can be used to assess muscle quantity and quality, challenges persist. The tools and skills required to quantify muscle on CT are not available in routine clinical practice. The GA, however, can and should be integrated into the care of older adults, as recommended by current ASCO guidelines [45]. The GA is particularly well‐suited to screen those at risk for sarcopenia and severe sarcopenia, as it captures essential muscle strength and physical performance data, as well as other factors closely associated with sarcopenia, such as ADLs, IADLs and recent falls. Although investigators are working to bring CT muscle assessments to the clinic, our findings suggest that the strength and performance measures obtained from a GA may help identify those older patients with cancer who may be at risk for severe treatment toxicity and poor cancer‐related outcomes. Innovative intervention trials that incorporate sarcopenia into treatment selection and treatment dosing are needed to advance this field further.

## Funding

This work was supported by the NCI UG1CA189961 (PI: K.M.M.), the NCI U01CA233167‐03S1 (PI: R.F.D.), the NCI OT2CA278664 (PI: K.M.M.) and the NCI T32CA102618 (Fellow: L.J.M.).

## Ethics Statement

Written informed consent was received from participants prior to inclusion in the trial. This study was approved by the Institutional Review Boards (IRBs) at all participating sites and has therefore been performed in accordance with the ethical standards laid down in the 1964 Declaration of Helsinki and its later amendments.

## Conflicts of Interest

R.F.D. has received honoraria from Eisai, Merck & Co. and Incyte for serving on an advisory board and honoraria for serving on an executive steering committee for Pfizer Inc. M.D.G. reports consulting or advisory roles with Almac Discovery, Genentech Inc., Scorpion Therapeutics, Skye Biosciences and Third Arc Bio; stock or other ownership interests in Faeth Therapeutics and Skye Biosciences; and patents, royalties and other intellectual property with Weill Cornell Medicine unrelated to the subject of this manuscript.

## NIH Public Access Statement

This work is the result of NIH funding, in whole or in part, and is subject to the NIH Public Access Policy. Through acceptance of this federal funding, the NIH has been given a right to make the work publicly available in PubMed Central.

## Supporting information


**Table S1:** Grades 3–5 nonhematologic toxicities according to sarcopenia (*N* = 161).
