# Supplementary material for: Sarcopenia Is Associated With Severe Treatment Toxicity and Mortality in Older Adults With Advanced Cancer: A Secondary Analysis
Source: J Cachexia Sarcopenia Muscle. 2026 Sep 24;17(5):e70388. doi: 10.1002/jcsm.70388 (PMC13602984; doi:10.1002/jcsm.70388)
Supplement: Supplementary file 1 [file JCSM-17-e70388-s001.pdf]

**Supplemental Table 1**: Grade 3-5 non-hematologic toxicities according to Sarcopenia (N=161)				
Toxicity	Non-Sarcopenia (n=70)	Sarcopenia (n=91)	Total (N=161)	p-value
Any Gr 3-5 non-heme tox	31 (44.3%)	58 (63.7%)	89 (55.3%)	**0.014***
Electrolyte Disturbance	5 (7.1%)	13 (14.3%)	18 (11.2%)	0.154*
Fatigue	4 (5.7%)	13 (14.3%)	17 (10.6%)	0.079*
Hypertension	6 (8.6%)	8 (8.8%)	14 (8.7%)	0.961*
Dehydration/Hypotension	6 (8.6%)	7 (7.7%)	13 (8.1%)	0.839*
hyperglycemia	3 (4.3%)	9 (9.9%)	12 (7.5%)	0.180*
Infection	5 (7.1%)	6 (6.6%)	11 (6.8%)	1.000**
Pain	2 (2.9%)	7 (7.7%)	9 (5.6%)	0.301**
Diarrhea/Colitis	1 (1.4%)	6 (6.6%)	7 (4.3%)	0.139**
Dyspnea/Hypoxia/ Resp Failure	4 (5.7%)	2 (2.2%)	6 (3.7%)	0.405**
Generalized Muscle Weakness	3 (4.3%)	2 (2.2%)	5 (3.1%)	0.654**
Nausea/Vomiting	1 (1.4%)	3 (3.3%)	4 (2.5%)	0.633**
Anorexia	1 (1.4%)	3 (3.3%)	4 (2.5%)	0.633**
LFT/Blood Bilirubin Increase	2 (2.9%)	2 (2.2%)	4 (2.5%)	1.000**
Syncope	2 (2.9%)	2 (2.2%)	4 (2.5%)	1.000**
Rash/pruritis	2 (2.9%)	2 (2.2%)	4 (2.5%)	1.000**
Hypoalbuminemia	2 (2.9%)	1 (1.1%)	3 (1.9%)	0.580**
GI Ulcer/Stomatitis	1 (1.4%)	1 (1.1%)	2 (1.2%)	1.000**
Bowel obstruction/Perf	0 (0.0%)	2 (2.2%)	2 (1.2%)	0.505**
GI Bleed	2 (2.9%)	0 (0.0%)	2 (1.2%)	0.188**
Thromboembolic Event	1 (1.4%)	1 (1.1%)	2 (1.2%)	1.000**
Fracture	1 (1.4%)	1 (1.1%)	2 (1.2%)	1.000**
Confusion/AMS	1 (1.4%)	1 (1.1%)	2 (1.2%)	1.000**
Limb edema/Ascites	0 (0.0%)	2 (2.2%)	2 (1.2%)	0.505**
Tumor Lysis	0 (0.0%)	1 (1.1%)	1 (0.6%)	1.000**
Wound Complication	0 (0.0%)	1 (1.1%)	1 (0.6%)	1.000**
Allergic Reaction	0 (0.0%)	1 (1.1%)	1 (0.6%)	1.000**
Pneumothorax	1 (1.4%)	0 (0.0%)	1 (0.6%)	0.435**
AKI/creat. Inc.	1 (1.4%)	0 (0.0%)	1 (0.6%)	0.435**
Neuropathy	0 (0.0%)	1 (1.1%)	1 (0.6%)	1.000**
Obesity	1 (1.4%)	0 (0.0%)	1 (0.6%)	0.435**
Constipation	1 (1.4%)	0 (0.0%)	1 (0.6%)	0.435**
Dysphagia	1 (1.4%)	0 (0.0%)	1 (0.6%)	0.435**
Stroke/visual disturbance	0 (0.0%)	1 (1.1%)	1 (0.6%)	1.000**
Fall	1 (1.4%)	0 (0.0%)	1 (0.6%)	0.435**

*Chi-squared test; **Fisher's exact test
